# ADDRESSING GAPS IN PRIMARY CARE DIAGNOSIS OF COGNITIVE IMPAIRMENT VIA A NURSE CONSULTATION

**DOI:** 10.1093/geroni/igac059.2022

**Published:** 2022-12-20

**Authors:** So Young Shin, Nhat Minh Bui, Rachel Kiekhofer, Collette Goode, Sarah Dulaney, Katherine Possin

**Affiliations:** Inje University, Busan, Pusan-jikhalsi, Republic of Korea; Memory and Aging Center University of California, San Francisco, San Francisco, California, United States; Memory and Aging Center University of California, San Francisco, San Francisco, California, United States; Memory and Aging Center University of California, San Francisco, San Francisco, California, United States; Memory and Aging Center University of California, San Francisco, San Francisco, California, United States; Memory and Aging Center University of California, San Francisco, San Francisco, California, United States

## Abstract

**Purpose:**

This study aimed to identify the concerns and unmet needs of patients and care partners after incident primary care diagnosis of cognitive impairment.

**Methods:**

Primary care providers referred older adults who had newly diagnosed with cognitive impairment for a telephone encounter, the ‘Brain Health Consultation’ (BHC), with a dementia expert nurse. The nurse assessed for questions or concerns regarding immediate needs, cognitive, neuropsychiatric, functional, or other symptoms; cognitive assessment results and the diagnosis; care planning including safety, prognosis, treatments, advance care planning, and community services.

**Results:**

Patients (N=37) and care partners (N=30) completed the BHC. The patients were racially/ethnically diverse; 51% Asian, 18% Non-Hispanic White, 10% Hispanic, 10% Black, 11% other). Most patients (70%) and caregivers (70%) endorsed cognitive concerns, and many patients endorsed mood (65%), sleep or fatigue (49%), and pain (10%) concerns. All patients and care partners had questions about the assessment results and diagnosis, and some patients (11%) and caregivers (13%) expressed concerns about disease progression. Few patients and caregivers expressed care planning needs.

**Conclusion:**

Following incident cognitive impairment diagnosis in primary care, patients and families have unmet needs around understanding their assessment and diagnosis. Care planning may be reserved for a follow-up consultation after the patient and family have had time to understand and accept the diagnosis. While we used a dementia expert nurse to perform the BHC, given the types of concerns identified, a supervised, trained, unlicensed health professional (e.g., a care team navigator) may be appropriate to perform the BHC.

